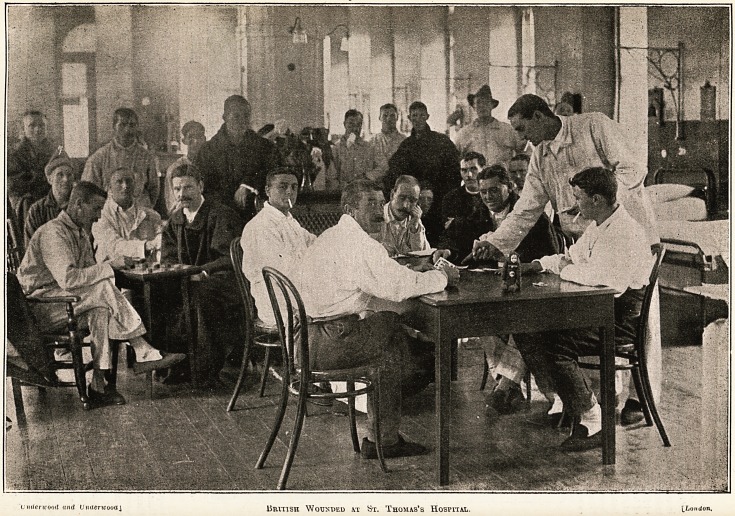# St. Thomas's Hospital as a Type

**Published:** 1915-06-12

**Authors:** 


### WAR-TIME AT A VOLUNTARY HOSPITAL.*

### St. Thomas's Hospital as a Type.

THE war has brought many changes in our lives, in our homes, and institutions, and nowhere has it been felt more than in the great voluntary hospitals of London. These have been affected in a multitude of ways. As might have been expected, the services of the distinguished members of the staff have been freely called upon for the benefit of the country. Nurses have been requisitioned for duty both in Navy and Army hospitals. Students have rushed off eagerly to serve their country, both qualified and unqualified men. Many of the lay staff just as enthusiastic have joined the combatant forces. With their reduced staffs all the hospitals have been called upon to undertake very heavy extra duty and to treat the wounded. It is invidious to single out any one hospital as doing more than others when all have done their best, but we may take St. Thomas's as a type and describe what has been done there in this grave time of emergency, and draw a very accurate conclusion as to what has been done, not only in the great hospitals in London, but in all similar institutions throughout the country.

*Underwood and Underwood*] BRITISH SOLDIERS IN A VOLUNTARY HOSPITAL WARD. [*London.*
(St. Thomas's Hospital.)

Immediately on the outbreak of war the Governors wrote on August 5 and offered to place at least 200 beds for non-commissioned officers and men and thirty beds for officers at the service of either Navy or Army. The offer was gladly accepted, and five wards on the first floor were set apart for the purpose. A meeting of the staff was held, and they cordially endorsed the action taken by the Governors. They even went further, and it was suggested amongst them that at least twice as many beds might be offered for this noble purpose. The Governors, however, felt that it was their duty first and foremost not to reduce to a point of grave injustice the number of beds allotted to civilian cases. Examining the matter carefully, they came to the conclusion that they should take two men's surgical wards, one men's medical ward, one female surgical ward, and one female medical ward. To some extent this could be done owing to the large number of men who are being drafted into the Army and the slight reduction resulting therefrom of the number of men applying for admission and

* These articles on pp. 233 to 238 are taken from our "Hospital Sunday Special Number," which is published, and may be obtained separately for distribution in the churches.

Underwood and Underwood] British Wounded at St. Thomas's Hospital. [London.

### War-time at a Voluntary Hospital.

treatment in the hospital. Further, under the system of allowance there is no doubt whatever that both women and children are better off. Better feeding at home and better living have undoubtedly reduced somewhat the number of cases of women applying for treatment in the hospital. The wards of St. Thomas's are fine, large, airy wards, with abundant cubic space per bed and excellent ventilation. So far, therefore, as space was concerned there was no reason why the usual thirty beds should not be increased by at least one-third, and in times of heavy pressure and emergency the numbers of patients admitted have been increased 30 per cent. This could not be done without the nursing staff, under the lead of the able matron of St. Thomas's, being prepared to bear their extra burden. They have been assisted by voluntary nurses, some with experience, some anxious to learn all that was possible and to be of use to those who are fighting for them.

On September 3 the first batch of wounded arrived, 123 men and one officer. They were practically all men who had taken part in the great retreat from Mons, and had been drafted over as soon as possible, shipped from Havre to Southampton, and brought thence by train to Waterloo, where they were met by the well-organised ambulances, under the direction of Mr. Clinton Dent, of the City Red Cross Ambulance Association. Amongst these men, however, very few were wounded; mostly, they were suffering from the extraordinary hardships of the early days of the campaign. One man with a bullet through his foot was quite a notable character, and he had shot himself by accident. Nothing showed how keenly the country was stirred more than the great number of strangers who applied for permission to visit these soldiers. There was very great risk that visitors inexperienced in hospital work would be a source of considerable danger amongst these men. Discipline was only maintained with very considerable difficulty. All were heroes, and hero-worship without judgment may be a little dangerous to the heroes. Many visitors, however, came to see these men; their presence cheered them and served to convey a message to those still fighting at the Front of how deeply the best of our land were stirred and were heart and soul in sympathy with those risking life and limb in their country's cause.

Their Majesties the King and Queen paid a semi-private visit to the hospital on September 8. Strict orders were given that none but the actual officials of the hospital should meet their Majesties. They wanted only the Secretary and the Matron and the physicians and surgeons in charge of the various wards to tell them of the cases. Their wishes were naturally strictly carried out, and their Majesties spoke individually to practically every patient in the wards. In those days men came back battered and torn by the turmoil of the strife, scarcely any had badges. These had been secured as a rule by their sympathetic French admirers, and in reply to his Majesty's "Where is your badge?" it was invariably "Souvenir, Sir." Their Majesties' visit was followed by practically all the Royal Princesses, by the Secretary of State for War, and other distinguished persons.

The hospital has since carried on its work until, in the first nine months of the war, a total of 211 officers and 1,371 non-commissioned officers and men have been treated in the wards of St. Thomas's. In the early days the cases were, as a rule, comparatively slight in character. Army regulations, however, led to their being retained in the hospital long after civilian cases of a similar type would have been discharged, but at last came the time when our armies moving up from the Aisne, and starting from Hazebrouck, met the Germans on the Belgian frontier. Then much more severe cases began to arrive. Boulogne was used very frequently as the port of embarkation for the hospital ships. Still, some came to Southampton; many of those who went through the first great battle of Ypres were hurried across, almost straight from the battlefield, *via* Boulogne and Folkestone, to Charing Cross, and so on to the hospital. Many arrived within three days of being wounded, and the demands on the services of the staff were proportionately heavy. All such demands, however, were readily met.

Early in February the Government became fully alive to the fact that much increased accommodation for wounded was required in London. They beat about for means of housing the wounded and of staffing them. The new King George Hospital had been devised by the Red Cross Society and made widely known through the columns of the *Times*. Little, however, has been put before the public of what a deal of extra work our hospitals have willingly undertaken. In some instances the staffs have been invited to take over the medical and surgical care of patients established in infirmaries converted to military hospitals or to improvise military hospitals in national schools and other similar places. On application being made to St. Thomas's from the office of the D.D.M.S. of London a suggestion was put forward at once from the Governors that great economy might be secured with increased efficiency if extra accommodation could be found within the hospital walls, and it was suggested that huts should be built in each of the quadrangles between the blocks. The suggestion was warmly welcomed, and at last, after going through the usual routine, authority was received on March 31 for the Governors to proceed with the erection of these huts at the price submitted, and the War Office would bear the expense of erection. Now, at the end of May, the building of these huts is completed, and progress has been made with their equipment. Already three huts are practically fit for use. There are six huts altogether for patients—two of sixty-six beds each, one of sixty beds, one of eighty beds, and two of thirty beds each. The two huts of thirty beds are what may be called single huts. The other four are divided by a spine 5 feet 6 inches high, with excellent cross ventilation, ample light, and are really charming wards. As may be imagined, there

### War-time at a Voluntary Hospital.

is a striking contrast between the hospital equipment and the War Office equipment, but the latter is at least serviceable, and we trust it will prove fully adequate to the end of the war. Each of these wards is provided with lavatories and bathrooms of the hospital type, each has its own little linen room, servery, and sister's small room. They are warmed by gas radiators, lighted by electricity, with abundant plugs for any hand-lights. In construction these wards, which are 104 feet long, 40 feet broad the double huts, 20 feet the single, 10 feet 6 inches high, are built of a wooden frame, outside asbestos slabs, inside fibrous plaster coloured with a pleasant light green. The window sashes open with a sort of fanlight, top and bottom. In addition, accommodation has been built for forty orderlies, for six sergeants, and a temporary extension of the kitchen has been made. The total cost is roughly £10,000.

When the Governors made their original offer they undertook to bear the full expense of the treatment of both officers and men, and this arrangement was maintained right through to March 1. The hospital resources were strained to almost breaking point, and when extra accommodation was asked for by the War Office they naturally looked for payment for maintenance. The agreement applies to all soldiers treated at the hospital, and the Governors will therefore receive payment at an agreed sum per diem from March 1. Though this amount is considerably less than the average cost of an ordinary hospital patient, it is felt that such help may be secured by the Governors in acknowledgment of the great work undertaken by them that the finances of the hospital may not be permanently impaired. Some donors have already recognised what is being done. Two American ladies in the early days of August expressed their anxiety to do everything in their power to help and to identify themselves with one hospital. Through one of the senior physicians of the hospital, Dr. Hector Mackenzie, these two ladies, Mrs. Cornelius Garrison and her sister, Miss Randell, sent a donation of £5,000 as a War Fund. That the men have appreciated their treatment is abundantly proved. Many friends of the hospital have generously sent supplies of cigarettes, tobacco, periodicals, flowers, and other gifts. The Colonial Governments have rendered very valuable aid by gifts of provisions, such as meat, fruit, flour, potatoes, etc.

One feature of the work must not be passed without mention. Quite a large number of young men who have applied for enlistment were found to be unsuitable unless they had an operation done for the cure of hernia, varicocele, and other small ailments. An average of something like forty per week have been taken in since the early days of the war. This could not have been done without generous aid from various convalescent homes and other institutions which have been specially prepared for the purpose. Lady Henry Somerset placed no fewer than fifty beds at Duxhurst at the disposal of the hospital. The Speaker gave timely aid at Wickham Market, and has taken a stream of patients—something like twenty—per week. Other homes have been opened by Mrs. Hamilton Grace, Mrs. Baxendale, and Mr. Lambert, and the great and generous work done deserves sincere thanks and appreciation from the nation.

A visit to the wards at St. Thomas's is convincing proof of the general happiness of the patients. The way in which the resident staff have risen to the occasion is worthy of all praise. They have generously undertaken an enormous amount of extra duty, and they have bravely stuck to their hospital work when their hearts led them to be away where they would love to be, with their colleagues abroad. They have still denied themselves and continued to do the routine hospital work, which cannot possibly be neglected.

## Figures and Tables

**Figure f1:**
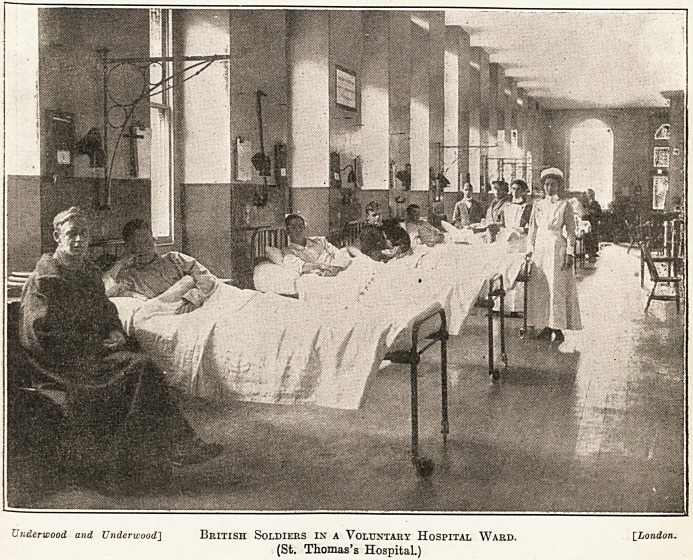


**Figure f2:**